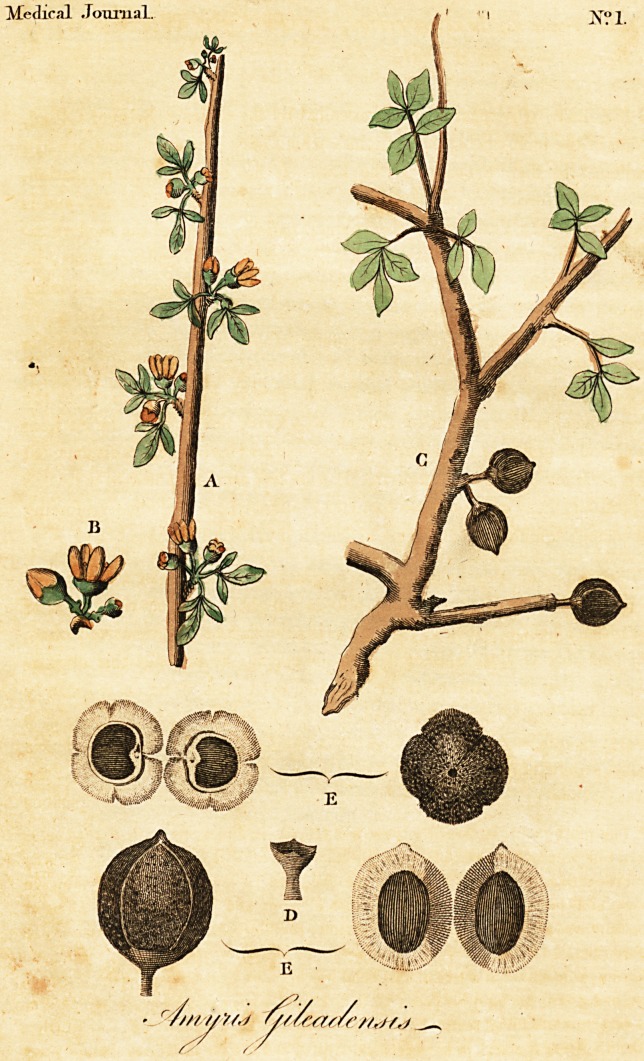# An Accurate Description of the Balm of Gilead; Its Growth, and Remarkable Properties

**Published:** 1799-03

**Authors:** 

**Affiliations:** Berlin


*Amyris Gileadensis*

### *An accurate Defcription of the Balm of Gilead; its Growth, and remarkable Properties, by Profeffor* WILDENOW, *of Berlin.—With a Plate.*

THE Balm of Mecca, or as it was called by the ancients, Balm of Gilead, is one of the moft celebrated medicines which have long been, and are ftill employed by the Phyficians of the Eaft. It has preferved its reputation, from a period prior to the birth of Chrift*, even to the prefent day, and is confidered by the Turks, and other oriental nations, as one of the moft efficacious and univerfal medical remedies. To whatever circumftance it may be afcribed, whether from the adulterations to which it is expofed, in paffing through fo many mercenary hands, before it can arrive on our fhores, or from the monopolizing fpirit of the Eaftern defpots, who carry on a profitable traffic with this highly efteemed balm, it is but rarely imported into Europe. In its genuine ftate it is fent from Mecca, now its native foil, only to the great princes and fovereigns of Europe, as a fcarce and valuable prefent from the Grand Seignior. In this ftate it is kept in fquare leaden bottles ornamented with a variety of whimfical figures. If it be frequently expofed to the accefs of frefh air, many of its volatile particles evaporate; and, gradually affuming a tenacious confiftence, it changes at length into a folid refinous body.

The odour of this balm, in its original ftate, refembles a compound of rofemary and fage, partaking alfo in a flight degree of the nature of turpentine; befides which it partially emits the flavour of lemons and mace. The beft fort is clear and of a greenifh colour; it poffeffes this fingular property, that if a drop of it be depofited on a glafs of water, it inftantly fpreads over the whole furface and may be eafily removed by means of a needle, having acquired the form of a tender pellicle. By this peculiar property the true Balm of Gilead may be readily diftinguifhed from all the fpurious

* The place where it formerly grew was Gilead, in Judea, more than 1730 years before Christ, or 1000 years before the Queen of Saba came to Jerusalem; and nothing is more certain, than that the balsam-tree had been transplanted from Abyssinia to Judea, and become an article of commerce there; and the place from which it originally was brought, through length of time, combined with other reasons, came to be forgotten. This is, however, contrary to the authority of JOSEPHUS, the Jewish historian, who says that a tree of this balm was brought to Jerusalem by the Queen of Saba, and given, among other presents, to Solomon, who, as we know from Scripture, was very studious of all sorts of plants, and skilful in the description and distinction of them. Here it seems to have been cultivated, and to have thriven so that the place of its origin came to be forgotten.——BRUCE's *Travels*, Vol. V.

ſpurious or adulterated kinds*; as it is a remarkable fact that very few of the oleaginous bodies exhibit a ſimilar phœnomenon.

The taſte of this balm is bitter, aſtringent, and acid. Among the Eaſtern nations it has long been a favourite and popular remedy taken internally, in caſes of diſeaſed inteſtines, ulcers of the lungs, liver, and kidneys; and, in general, it is reputed an excellent diaphoretic and alexipharmic medicine. To perſons who have ſwallowed poiſon, or have unfortunately been bit by ſerpents, ſcorpions, or other venemous animals, it is adminiſtered internally, as well as applied externally to the injured part. The modern Egyptians make daily uſe of it, during the ravages of the plague, in order to prevent or repel that deſtructive malady. It is further believed that the Egyptian women poſſeſs the wonderful art of rendering themſelves fruitful, either by the internal uſe of this balm, or by perfuming and ſmoking their bodies with it. The beauty of the ſkin is alſo ſaid to be not a little improved by the uſe of it; and the ladies of the ſeraglio anoint their bodies with it, after tepid bathing. A celebrated Engliſh lady who long reſided in Turkey, ventured to imitate this oriental cuſtom; but, whether from a too prodigal uſe of the balm, or the improper application of it, diſagreeable effects followed; her face ſwelled violently, the whole of the epidermis, or ſcarf-ſkin, peeled off, and her whole body broke out in blotches, and other cutaneous eruptions. Throughout the Eaſt, the balm of Mecca is to this day conſidered as a ſovereign remedy againſt all diſeaſes; and ſuch is the unalterable veneration the Orientals entertain for it, that every part of this tree is, in ſome form or other, converted to medical purpoſes. For, beſides the balſam or balm, its fruit is employed under the name of what we call in Europe *Carpobalſamum*, and the wood, ſtalk or trunk, under that of *Xylobalſamum*.

The ancient oriental phyſicians made uſe only of that balm which ſpontaneouſly dropped from the tree, or which exuded after inciſion: at preſent, however, there are *three* different methods of obtaining it, each of which furniſhes a diſtinct ſpecies of the balm. The firſt mode of collecting it, is that

* When Sultan Selim made the conqueſt of Egypt and Arabia, in 1516, three pounds was then the tribute ordered to be ſent to Conſtantinople yearly; and this proportion is kept up to this day. One pound is due to the Governor of Cairo, one pound to the Emir Hadje, who conducts the pilgrims to Mecca, half a pound to the Baſhaw of Damaſcus, and ſeveral ſmaller quantities to other officers; after which the remainder is ſold or farmed out to ſome merchants, who, to increaſe the quantity, adulterate it with oil of olives, and wax, and ſeveral other mixtures, conſulting only the agreement of colour, without conſidering the aptitude of mixing. Formerly, we were told, it was done with art; but nothing is eaſier detected than this fraud now.
*Bruce's Travels*, Vol. V.

that pursued by the ancients, or by incision, which produces by far the most valuable balm; this species is never exported as an article of trade, and its consumption is chiefly confined to the principal and richest families of Mecca and Constantinople. The second mode of producing the balm depends upon boiling the branches and leaves: this sort is perfectly pellucid, and emits an agreeable fragrance. The Turkish ladies apply it externally to beautify their skin, and make the hair grow; this is the kind which the Grand Seignior sometimes sends as a present to other princes, and which is occasionally vended in the shops, as a rare and costly article. The last, and a very inferior species of the balm, is obtained by a repeated and stronger decoction of the leaves and branches: in this state it becomes much thicker, but less fragrant, and is transported to Europe by the caravans, under the different names of balm of Mecca, Gilead, Judea*, or *Opobalsamum*, signifying the juice of the tree. This kind is not much valued in the East, and is used only by the lower classes of people.

It appears from the most authentic ancient writings, that the Balm of Gilead was an important article of commerce, several centuries before the Christian æra; but, notwithstanding its well-founded claim to antiquity, its native soil (the place at least of its present growth) has been discovered only by the travellers of modern times. BRUCE, the Abyssinian traveller, has first pointed out the native soil of this balm, being the same as that of the myrtle, behind *Azab*, along the coast of Arabia, and extending to the Straits of *Babelmandel*. Anciently it was believed that Egypt, Palestine, and Arabia produced this balsamic tree; but, however that might be in former times, it is certain that now it is only cultivated artificially in those countries, that it does not thrive so well there as other indigenous plants, and that the inhabitants are obliged to import annually a fresh stock of young trees, to supply the place of the decayed ones.

Ancient writers have related many marvellous and fabulous things as connected with the history of this tree. Some asserted that vipers were continually breeding under its shade; according to others, it possessed such a degree of antipathy to iron, that it sensibly trembled, on the smallest particle of iron entering into contact with it, and that on this account any incisions made in its rind must be performed with ivory, glass, or some other hard substance. Mr. Bruce, however, was an eye-witness to this incision being

* The Balsam of Judea was long ago lost, when the troubles of that country withdrew the royal attention from it; yet, as late as GALEN's time, it not only existed, but was growing in many places of Palestine, besides Jericho, but there is no doubt that it is now totally lost there.——*Bruce's Travels*, Vol. V.

being made with an axe, without any trembling on the part of the tree, and it is alſo probable that ſimilar operations have been always made with the ſame inſtrument. Other writers have maintained, that perſons who anoint their bodies with this balm, have a peculiar claim to never-fading beauty, and to perpetual youth.

Of more importance to the botaniſt than theſe particulars, is the knowledge of the plant itſelf. LINNÆUS, in his Syſtem of plants, notices two ſpecies of the Amyris, namely the *Amyris Gileadenſis*, and *Opobalſamum*, both of which are repreſented in general terms as trees producing this balſam. The late Dr. GLEDITSCH, of Berlin, an eminent phyſician and botaniſt, had an opportunity of examining a branch, which he found to correſpond in its principal characters with the *Amyris Opobalſamum* of Linnæus. He diſcovered, however, in the ſtamina of the flower, deviations which appeared to him ſufficiently important to warrant the deſcription of it as a diſtinct genus; he accordingly gave it the name of *Balſamea Meccanenſis*. The difference in the parts of the flower, as obſerved by Gleditſch, conſiſted in the peculiar connection of the filaments, which, according to Linnæus's mode of defining it, are conſolidated with the thalamus, or receptacle; but which, in the plant examined by Gleditſch, were, in fact, connected with the bottom of the calyx.

The ſituation, or rather the manner in which the filaments are united to the flower, is a point of great importance in botany; for, although plants ſhould ſufficiently correſpond in all other parts of the flower, they muſt be conſidered as diſtinct genera, if a difference prevail in this reſpect. It is, however, frequently a matter of difficulty to aſcertain the true poſition of the filaments; becauſe the boundary between the calyx and the receptacle cannot, in all inſtances, be accurately diſtinguiſhed. After a diffuſe deſcription of the balm-tree, Linnæus informs us that the filaments are joined to the edge of the receptacle; while Gleditſch inſiſts that this edge is a part of the calyx itſelf. Hence it is evident, that the plant deſcribed by the latter naturaliſt does not in reality form a diſtinct genus, but is the ſame as that which Linnæus has given under the name of *Amyris*.

Although it be now certain that the balm-tree falls under the generical term of *Amyris*, it ſtill remains to be determined, whether it be the ſpecies called by Linnæus *Gileadenſis*, or that called *Opobalſamum*. In this reſpect, alſo, our doubts will diſappear, if we compare the hiſtorical notices which we have of the plant, with the accounts of its character given by botaniſts. Linnæus deſcribes the *Amyris Gileadenſis* as a plant with ternate leaves, (*foliis ternatis*) which are not indented on the edge: the *Amyris Opobalſamum*, on on the contrary, he characterises as having pinnated leaves, *(folia pinnata)* the leaflets of which are without a stem. In the specimen of the plant observed by Gleditsch, were double pinnated leaves *(folia bipinnata)*. This difference in the accounts might lead us to conclude, that there existed a distinction of three different balm-trees; but if we attentively compare the manner in which different, and particularly kindred plants, proceed in their vegetation, these three apparently distinct plants will prove to be manifestly one and the same species. Indeed most plants are, in their different stages of growth, subject to a variety of changes. Those which, in their infant state, usually have simple leaves, with their increasing age have them more compounded; and *vice versa*. The latter is, for instance, the case in the family of plants called by botanists *Lomentaceä*, and which includes two genera, distinguished by the names of *Gleditsia* and *Mimosa*. The well-known *Gleditsia triacanthos*, when young, has double, and sometimes triple pinnated leaves, which, as the plant advances in age, become gradually less compounded, till at length they degenerate into simple pinnated leaves. Certain species of the genus *Mimosa*, lately discovered on the coast of New Holland, and which differ from all others hitherto known with simple leaves, shew in their infancy very compounded double pinnated leaves. The *Amyris*, therefore, appears to be nearly related to the family of *Lomentaceä;* and hence it may be reasonably conjectured, that in some of the species pertaining to the former, the leaves may, in their infant state, be more compounded than in others.

Upon comparing the delineations of this plant, as given by Prosper Alpinus, and by Mr. Bruce, in the fifth volume of his Travels, it seems difficult to point out any perceptible difference between them. The former, in his "*History of the Plants of Egypt*," furnishes us with a plate representing a figure, the lower leaves of which are ternate, and the upper ones pinnate; these last again are compounded partly of five, partly of seven leaflets. The drawing given by Mr. Bruce is incomparably better and more accurate, although the leaves are not exhibited with uniformity, as they are sometimes ternate, and in other places pinnate. The pinnate leaves throughout consist of five leaflets only, in all other respects, the single pinnate leaves excepted, this representation of the *Amyris Gileadensis* agrees very exactly with one published by Professor Vahl, in his "*Symbolæ Botanicæ.*" It should be remarked, however, that Mr. Bruce has seen a full-grown tree, and from the figure given by Alpinus, it is highly probable that he was acquainted only with young trees of this kind. Forskal, another botanical writer, from whose collection of plants Professor Vahl borrowed his representation of the balm-tree, had made his drawing perhaps from an old tree. The plant from which Gleditsch was enabled to furnish his account and delineation, was probably a very young one, one, and had been collected in the earlier years of its growth. Upon the whole, the diſſimilar deſcriptions and repreſentations of this plant given by naturaliſts, may be ſo far reconciled as to juſtify the conjecture, that the *Amyris Gileadenſis* and the *Opobalſamum* of Linnæus are not two diſtinct genera or ſpecies, but that they are virtually ſpecimens of the ſame plant, for which the former may ſerve as the generic name.

To illuſtrate the repreſentation here annexed of the *Amyris*, it will be proper to accompany it with an accurate deſcription. According to Mr. Bruce's ſpecimen, it is a tree of an inferior ſize, not unlike a ſmall cherry-tree; its heighth from the root to the crown, or expanſion of the branches, being only five feet two inches, and the diameter of the trunk not exceeding five inches. The branches ſpread to a conſiderable diſtance around it, yet the crown, in general, is not much higher than the trunk. The root is of a red colour; the bark of a browniſh gray, ſomewhat like the young branches of a cherry-tree. Its wood is light, and of a looſe texture, ſo that it will not admit of poliſhing; and, in many reſpects, it reſembles the wood of our common willow. The tree itſelf preſents no very agreeable appearance, as the wide-extended branches make the crown of it look ſtunted and flat at the top, as is frequently obſerved in trees on our coaſts, expoſed to ſea-ſtorms, or violent northerly winds.

The leaves are ſmall, and ſituated cloſe to the branches; a circumſtance which gives the tree a naked appearance, although in reality it abounds in foliage*. The leaves themſelves are ſometimes ternate, ſometimes pinnate. On the young branches, between the leaves, there appear ſingle flowers, and occaſionally two or three white flowers, with ſhort ſtalks, cloſe to each other.

The

* Mr. Bruce, on the contrary, asserts that "it is remarkable for a penury of leaves;" and likewise maintains that "*a part of the bark is of a reddish brown*," after having said, immediately before this limitation, that "the wood of this tree is covered with a smooth *bark of blueish white*, like to a standard cherry-tree," &c.

Indeed, we need not wonder at such little inconsistencies, when we read the following confused and imperfect description of the parts of fructification, which we quote *verbatim* from Mr. Bruce's *Travels*: "The flowers," says he, "are like that of the accacia-tree, white and round, only that three hang upon three filaments or stalks, where the accacia has but one. Two of these flowers fall off, and leave a single fruit; the branches that bear this are the shoots of the present year; they are of a reddish colour, and tougher than the old wood: it is these that are cut off, put into little faggots, and sent to Venice for the Theriac, when bruised or drawn by fire; and formerly these made the *Xylobalsamum*."——*Bruce's Travels*, Vol. V.

The calyx is undivided, campanulous, four times indented, and remains till the fruit is ripe. At the bottom of the calyx, clofe to the receptacle, there appears a yellow ring, which, according to fome writers, is the nectarium. The corolla is polypetalous, the leaflets are of a linear form, and incline towards each other. It has eight ftamina, and the filaments are fhorter than the corolla, capillary, fomewhat bent, and are fituated on the yellow ring which appears at the bottom of the calyx. The anthers are double, of a yellow colour, and oblong. The receptacle is inclofed within the calyx, and remarkably fmall; the ftem is fhort and diminutive; the cicatrices or impreffions blunt and fquare. The fruit (*drupa*) is of a juicy nature, being about the fize of a goofeberry, and marked externally with four fmall furrows. It is filled with a tough vifcous juice, and contains a nut which inclofes one kernel of feed only.

Confiderable deviations are frequently obferved in the number of its parts; thus, for inftance, the calyx has been found five times indented; alfo, five petals or leaflets have been noticed, and ten ftamina.

EXPLANATION OF THE PLATE.

(A) A fmall branch of the *Amyris Gileadenfis*, nearly in its natural fize. (B) The flower fomewhat magnified. (C) A fpecimen of a branch with fruit in its unripe ftate. (D) The Calyx. (E) The fruit in its natural fize, with tranfverfe and longitudinal fections.

## Figures and Tables

**Figure f1:**